# Determination of Tryptophan and Its Major Metabolites in Fluid from the Anterior Chamber of the Eye in Diabetic Patients with Cataract by Liquid Chromotography Mass Spectrometry (LC-MS/MS)

**DOI:** 10.3390/molecules23113012

**Published:** 2018-11-17

**Authors:** Jolanta Flieger, Anna Święch-Zubilewicz, Tomasz Śniegocki, Joanna Dolar-Szczasny, Magdalena Pizoń

**Affiliations:** 1 Department of Analytical Chemistry, Medical University of Lublin, Chodźki 4A, 20-093 Lublin, Poland; magdalenapizon@umlub.pl; 2 Department of Retinal and Vitreal Surgery, Medical University of Lublin, Chmielna 1, 20-079 Lublin, Poland; annazub@umlub.pl (A.Ś.-Z.); joannaszczasny@op.pl (J.D.-S.); 3 Department of Pharmacology and Toxicology, National Veterinary Research Institute, 24-100 Pulawy, Poland; sniego@piwet.pulawy.pl

**Keywords:** chromatography, tryptophan metabolites, kynurenine pathway, cataracts, diabetes

## Abstract

Tryptophan (TRP) is to an essential amino acid and its catabolites are significant to human health. By using ultra-high-performance liquid chromatography coupled to electrospray ionization triple quadrupole mass spectrometry (UHPLC-ESI-MS/MS), levels of three major components of kynurenic pathway namely tryptophan (TRP), kynurenic acid (KYNA) and kynurenine (KYN) in fluid from the anterior chamber of the eye were determined. The analysis was carried out on a Synergi 4 μ Fusion-RP column using gradient elution mode. For quantitative determination, l-tryptophan-amino-15N, 99 ATOM % 15N was used as an internal standard. The method was linear in the concentration range 4–2000 ng mL^−1^ for TRP, KYNA and KYN. The mean recoveries measured at four concentration levels for TRP, KYN and KYNA included the following ranges 94.3–96.1; 91.0–95.0; and 96.0–97.6%, respectively. The intra-day precision parameters were smaller than 4.4, 6.4 and 5% respectively. The developed method was applied to study the level of TRP, KYNA and KYN in eye fluid for the retrospective case series which included 28 patients suffering from cataracts and diabetes (*n* = 8). The experimental data was subjected to statistical analysis. The Mann-Whitney U-test revealed clear differences in the level of TRP catabolites and the ratios of TRP/KYN representing the activities of specific enzyme of kynurenine pathway in examined groups of patients. A level of probability *p* < 0.05 was used throughout a paper to denote statistically significant differences between the groups.

## 1. Introduction

The main route of tryptophan (TRP) degradation is through kynurenine pathway (KP) accounting for ~95% of its overall degradation. The KP leads to production of metabolites, such as kynurenic acid (KYNA), kynurenine (KYN), anthranilic acid (AA), 3-OH kynurenine (3-OH KYN), xanthurenic acid (XA), 3-hydroxybutyrate anthranilic acid (3-HAA), quinolinic acid (QA) subsequently denoted as kynurenines and, finally, to biosynthesis of a crucial cofactor, namely, nicotinamide adenine dinucleotide (NAD+) [1]. The process of metabolizing TRP into KYN takes place in different locations mostly in the liver, kidneys and the brain with the help of three enzymes: TRP-2,3-dioxygenase, indoleamine-2,3-dioxygenase-1 and indoleamine dioxygenase—2 [1,2]. It should be emphasized that humans are not able to synthesize TRP by themselves, therefore, it comes only from the diet. In recent years, many researches have proven a significant role of the KP in the pathogenesis of several serious disorders, such as cancer, psychiatric, neurodegenerative and autoimmune system dysfunctions [3]. The examination of TRP metabolites levels has been studied in brain tissue and amniotic fluid [4], serum [5,6] cerebrospinal fluid [7], human cataracts’ lenses [8] and saliva of a diabetic patient [9]. There is also evidence that the KP pathway is connected with visual function as KYN and 3-OH-KYN glycoside derivatives act as UV filters and protect the retina and the lens by absorbing the UV radiation [8,10,11,12,13,14]. On the other hand, the kynurenine pathway-derivatives were identified as compounds inducing that is, cataract formation [8,10,14]. Till now determination of TRP catabolites has been developed by a sensitive, rapid and specific high-performance liquid chromatography coupled to tandem mass spectrometry, however, ultra-violet or fluorescent absorbance and electrochemical detectors can be also applied for this purpose [15,16,17,18,19,20,21,22,23,24,25].

The goal of this study was to quantify of tryptophan and its major metabolites, kynurenine and kynurenic acid by LC-MS/MS using l-tryptophan-amino-15N, 99 ATOM % 15N as an internal standard. The elaborated method was assessed for validation parameters. Feasibility of the method was demonstrated in fluid from the anterior chamber of the eye in patients. Patients were divided into those with cataracts and compared to patients with coexisting diabetes. To test differences between the groups, the experimental results have been analysed by a statistical method. The levels of TRP, KYN, KYNA, the ratios of catabolites to each other were considered as the factors. Additionally, the age of patients was considered as an independent variable.

## 2. Results and Discussion

LC/MS/MS as an essential method to identify and quantify TRP and its kynurenine metabolites was applied to the present study. Our procedure was based on a simple “dilute-and-shoot” liquid chromatography-tandem mass spectrometry (LC-MS/MS) method which was previously used for the simultaneous quantitation of drugs of abuse, in biological samples for forensic toxicology purposes [26,27]. UHPLC ensured and improved either throughput and sensitivity of the method.

**Figure 1:**
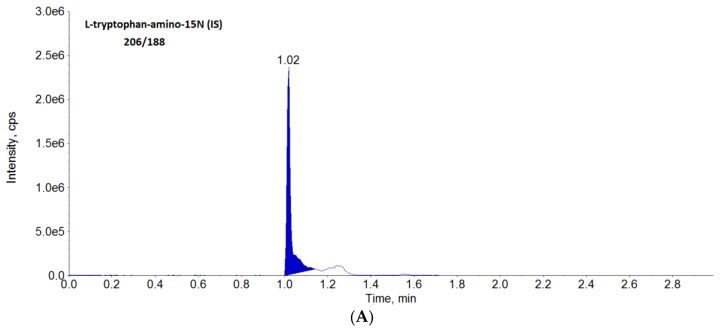

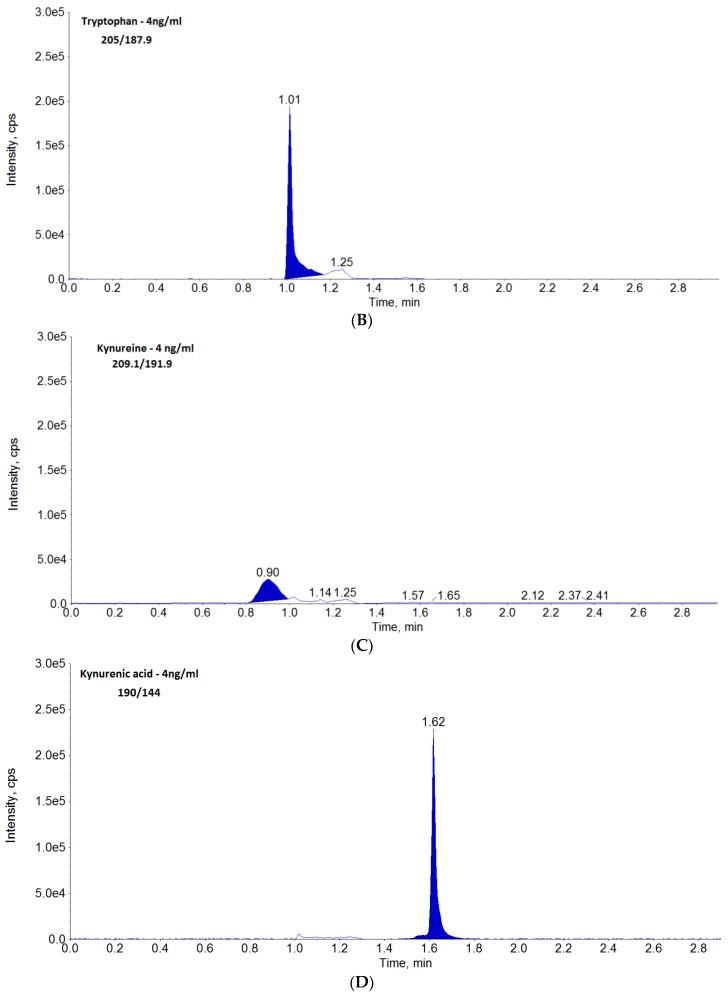
Chromatograms obtained from analysis of standards of IS (**A**), tryptophan (**B**), kynurenine (**C**), kynurenic acid (**D**) by LC-MS/MS.

The elaborated conditions ensured successful separation of examined analytes within 2 min (Figure 1).

Proposed procedure was subjected to a number of validation criteria as, so far, there is no studies describing analysis of TRP metabolites in the fluid from the anterior chamber of the eye. A linear response of the peak area was observed over a concentration range of 4–2000 ng mL^−1^. It should be emphasized that such a wide range of the calibration curve from 4–2000 is acceptable for API instrument [28,29]. The correlation coefficient values obtained from plotting the peak area against the nominal concentration were higher than 0.99 at each case. Linearity was observed for each concentration range between this at the LOD and the highest concentrations tested. The parameters of the linear regression function are collected in Table 1.

LOD and LOQ were calculated as the analyte concentrations which give rise to peaks 3 and 10 times higher than the baseline noise, respectively, varied between 0.2 and 1.20 ng/mL for LOD and 0.66 and 3.9 ng/mL for LOQ (Table 2).

The relative recoveries were estimated by measuring spiked samples of TRP, KYN, KYNA at four concentrations with 3 replicates of each. No statistically significant differences were noticed for the lower and higher concentrations as shown in Table 3. Because the fluid from the anterior chamber of the eye without tryptophan and its metabolites does not exist, purified water was used as a matrix. As shown, recoveries of TRP, KYN and KYNA varied between 94.3–96.1%, 91–95% and 96–97.6% respectively.

The method provided satisfactory precision expressed by relative standard deviation values. Precision values expressed as RSD% values were lower than 5% for repeatability (intra-day precision) and lower than 7% for intermediate precision (reproducibility, inter-day precision).

The results in Table 4 show content of the kynurenine metabolites determined in aqueous humour of 28 subjects who underwent cataract extraction surgery. All clinical samples have been divided into two groups: the first group including patients with cataract and the second one with cataract with coexisting diabetes.

The concentrations of KYN and KYNA were significantly higher in patients with diabetes in comparison to cataracts alone. There were no differences in concentration of TRP and other metabolites ratios between the studied groups. The increased concentrations of KYN and KYNA in patients with diabetes suggest an altered metabolism of TRP.

## 3. Materials and Methods

### 3.1. Sample Collection

Performed experiments were done in agreement obtained by the Bioethics Commission of the Medical University of Lublin. Twenty-eight samples of fluid from the anterior chamber of the eye were collected at the beginning of cataract extraction surgery. The samples were stored in 1.5 mL polypropylene tubes at −80 °C until analysis.

### 3.2. Sample Preparation

The purification was based on Dilute and Shoot (D&S) method. Briefly, 15 μL of the fluid from the anterior chamber of the eye was diluted by 15 μL of internal standards (l-tryptophan-amino-15N) and 30 μL of 0.1% formic acid in water. Then, 10 μL was injected into LC-MS/MS.

### 3.3. LC-MS/MS 

The UHPLC-MS/MS system consisting of an AB Sciex ExionLC UHPLC system connected to an AB Sciex API 5500 Qtrap mass spectrometer (AB Sciex, Concord, ON, Canada). Analyst 1.6.3 software (AB Sciex) controlled the UHPLC-MS/MS system and Multiquant 3.2 (AB Sciex) was used to process the data. The mass spectrometer was operated in the positive ESI mode with a capillary voltage of 4.5 kV. The temperature of desolvation was set at 600 °C, nebuliser gas (N_2_)–40; curtain gas (N_2_)–40; collision gas (N_2_)–medium; gas 1 (air)–35; gas 2 (air)–35. The multiplier was set at 2300 V. The flow rate of mobile phase was 600 μL min^−1^, the injection volume–10 μL. The chromatography was performed on a Synergi 4 μ Fusion-RP column (50 mm × 2 mm × 4 μm), connected to a C18 precolumn (4 mm × 2 mm × 4 μm). The mobile phase for LC analysis consisted of two solutions: A (0.1% formic acid in water) and B (acetonitrile). The mobile phase gradient program started at 1% of B, 98% B at 0.55 min to 1 min, then 1% of B at 1.1 min and held for 1.9 min. The column was equilibrated for 2 min. The column operated at 40 °C and the ions were monitored in Multiple Reaction Monitoring (MRM) mode (Table 6).

### 3.4. Validation

The analytical-method validation was carried out according to the ICH Q2 (R1) method-validation guidelines [30] and others [31,32,33]. The following validation parameters were established: selectivity, linearity, precision, LOD and LOQ. The method validation was also based on other literature data, describing an analysis of endogenous compounds in a biological matrix [34,35].

#### 3.4.1. Calibration Solutions and Standards

Analyte standard solutions at different concentrations: 4, 10, 50, 150, 400, 2000 ng/mL were added to the blank sample, subjected to the D&S and HPLC procedure. The analyte peak area was plotted against the corresponding concentrations and the calibration curves were set up by means of the least-squares method.

#### 3.4.2. LOD and LOQ

LOD and LOQ were estimated by calculations based on Signal-to-Noise ratio. Determination of the signal-to-noise ratio is performed by comparing measured signals from samples with known low concentrations of analyte with those of blank samples and establishing the minimum concentration at which the analyte can be reliably detected or quantified concentration at which the analyte can be reliably quantified. A typical signal-to-noise ratio is 3:1 for LOD and 10:1 LOQ.

#### 3.4.3. Precision

Spiked blank samples were prepared as follows: 15 μL of standard solution of different concentration were added to 15 μL of the water and was diluted by 15 μL of internal standards (l-tryptophan-amino-15N) and 15 μL of 0.1% formic acid in water. At least four concentrations were prepared and analysed for each compound, corresponding to the lower, two middle and upper limit of the respective linearity curve. Spiked blank samples were prepared and analysed according to the described procedure. The analysis was repeated twelve times giving intraday precision values and twelve times in another day giving intermediate precision values, both expressed as RSD%.

### 3.5. Statistical Analysis 

The statistical analysis was carried out in the STATISTICA versus 12 program using the Mann-Whitney U-test. The statistical significance level was assumed for *p* values <0.05.

## 4. Conclusions

Examination of tryptophan metabolism can help to understand an aetiology as well as consequences of many diseases. Experiments on the fluid from the anterior chamber of the eye suggest that neuroactive metabolites of the tryptophan (TRP) in the kynurenine pathway (KP) may play a significant role in cataract formation in patients suffering from diabetes. The elevation of KYNA levels has been previously observed in cataractous lenses [8]. Our study has proven the increased content of KYN and KYNA and the ratio of TRP/KYN in the eye fluid of cataract patients suffering from diabetes. Obtained results, similarly to literature data, confirm that tryptophan metabolism is not only dysregulated during diabetes but also affects ophthalmological complications.

However, there is a need for further experiments in the aim to support the obtained results and fully explain the role of tryptophan metabolism in the pathogenesis of cataracts in diabetic patients.

## Figures and Tables

**Figure 2:**
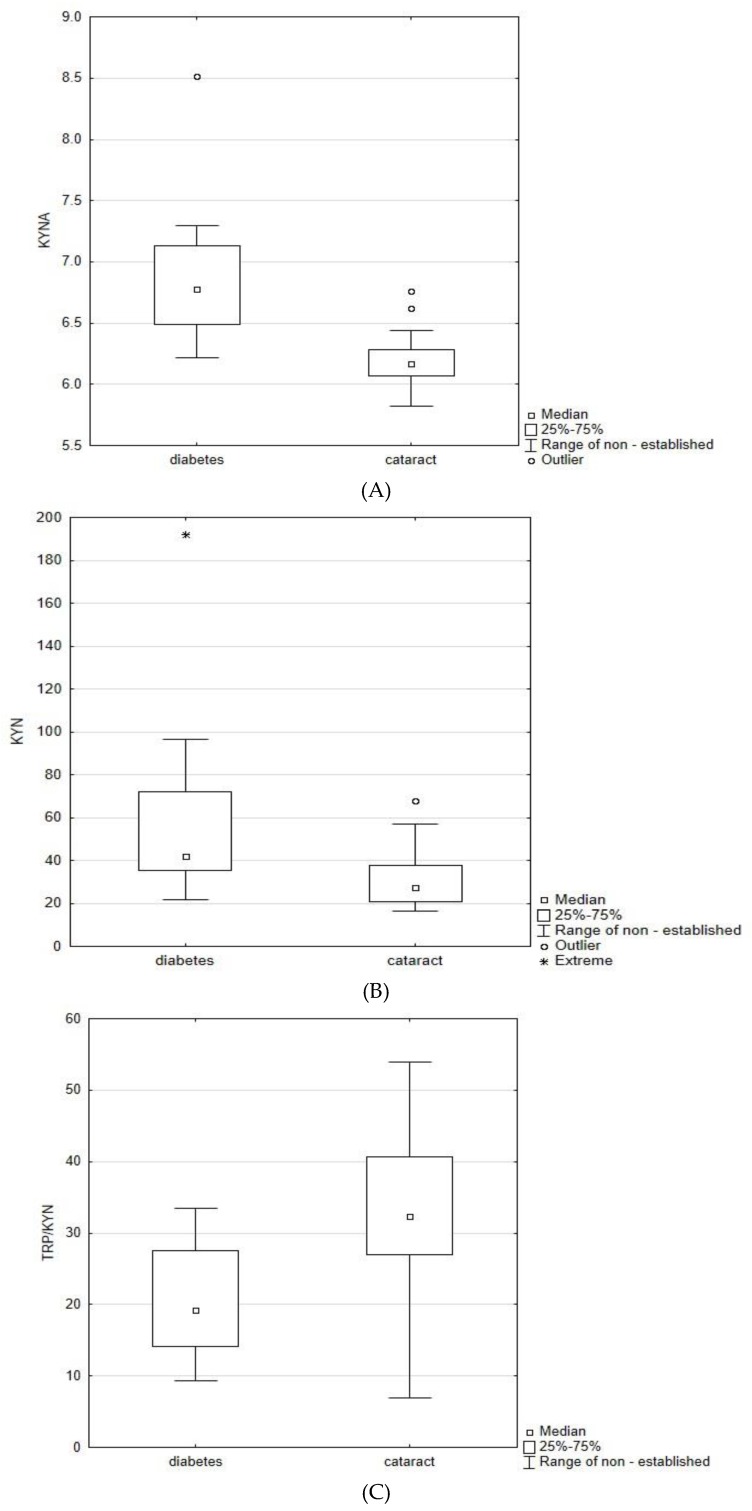
The box-plots representing statistically significant differences between the levels of KYNA (**A**), KYN (**B**) and ratio TRP/KYN (**C**) in patients with both studied groups.

**Table 1:** Parameters obtained for the calibration curve.

Analyte	Concentration Range (ng/mL)	Determination Coefficient	Calibration Curve
Tryptophan	4–2000	0.9973	y = 0.0213 (±0.0015)x − 0.0330 (±0.0023)
Kynurenine	4–2000	0.9972	y = 0.0325 (±0.0030)x − 0.0325 (±0.0030)
Kynurenic acid	4–2000	0.9982	y = 0.0088 (±0.0010)x − 0.1097 (±0.0121)

**Table 2:** LOQ and LOD values.

Analyte	Tryptophan	Kynurenine	Kynurenic Acid
LOD (ng/mL)	0.20	1.20	0.40
LOQ (ng/mL)	0.66	3.9	1.32

**Table 3:** Validation parameters.

**Parameters**	Tryptophan			
Fortification level (ng/mL)	50	150	400	2000
Average recovery (*n* = 18) (%)	95.0 ± 3.2	96.1 ± 3.4	98.0 ± 4.0	94.3 ± 2.2
Average repeatability, (RSDr, %)	4.4 ± 3.4	3.4 ± 2.2	3.6 ± 3.9	4.1 ± 4.1
Average within-lab reproducibility, (RSDwR, %)	5.7 ± 3.2	5.0 ± 3.0	5.2 ± 2.0	5.4 ± 3.8
	Kynurenine			
Fortification level (ng/mL)	10	50	150	400
Average recovery (*n* = 18) (%)	91.0 ± 4.6	92.2 ± 4.2	94.6 ± 3.0	95.0 ± 4.3
Average repeatability, (RSDr, %)	5.5 ± 3.9	4.9 ± 4.4	5.1 ± 4.3	6.4 ± 4.1
Average within-lab reproducibility, (RSDwR, %)	6.9 ± 5.3	5.1 ± 4.3	4.8 ± 4.5	5.8 ± 5.2
	Kynurenic acid			
Fortification level (ng/mL)	4	10	50	150
Average recovery (*n* = 18) (%)	96.0 ± 3.7	96.3 ± 3.5	97.2 ± 3.8	97.6 ± 2.6
Average repeatability, (RSDr, %)	5.0 ± 3.6	3.6 ± 2.9	3.2 ± 3.4	4.0 ± 3.7
Average within-lab reproducibility, (RSDwR, %)	5.3 ± 3.9	4.2 ± 3.3	4.4 ± 2.9	4.6 ± 3.2

**Table 4:** The level of TRP, KYNA and KYN [ng/mL] and metabolites ratios determined in eye fluid for the retrospective case series of 28 patients suffered from cataracts including 8 patients with coexisting diabetes.

	**Diabetes and Cataract (*n* = 8)**			
	**Range**	**Mean ± Standard Deviation**	**Median**	**CV**
KYN (ng/mL)	21.85–191.84	64.06 ± 56.17	41.93	0.876
KYNA (ng/mL)	6.22–8.51	6.94 ± 0.72	6.78	0.104
TRP (ng/mL)	694.86–1784.12	1010.69 ± 387.45	817.76	0.383
TRP/KYNA	101.57–256.80	145.53 ± 53.36	125.81	0.367
TRP/KYN	9.33–33.46	20.59 ± 8.72	19.23	0.423
KYN/KYNA	3.40–27.54	9.29 ± 8.12	6.51	0.874
	**Cataract (n = 20)**			
	**Range**	**Mean ± Standard Deviation**	**Median**	**CV**
KYN (ng/mL)	16.61–67.84	31.11 ± 14.59	27.37	0.469
KYNA (ng/mL)	5.83–6.76	6.19 ± 0.23	6.17	0.038
TRP (ng/mL)	465.36–1356.26	855.57 ± 203.13	835.02	0.237
TRP/KYNA	75.72–219.30	138.03 ± 31.00	132.25	0.225
TRP/KYN	6.86–53.91	32.12 ± 11.72	32.36	0.365
KYN/KYNA	2.71–11.03	5.01 ± 2.29	4.42	0.457

Data was presented as mean ± standard deviation and was all tested for homogeneity of variance by the Bartlett test at first. It appeared that obtained data did not follow the normal distribution. To compare two independent data coming from distinct populations, which can not affect each other, namely, “cataract group” and “diabetes group”, the Manna–Whitney-U test corrected for ties has been applied (Table 5). Besides the content of metabolites, data expressing the following ratios TRP/KYNA, TRP/KYN, KYN/KYNA, has been added as an additional variable. Obtained values of *p* indicate existence of the significant differences between “cataract group” and “diabetes group” regarding the level of KYN, KYNA and the ratio of TRP/KYN (Table 5, Figure 2). We did not find any correlations between remaining estimated parameters.

**Table 5:** The Manna–Whitney-U test corrected for ties. Marked tests are significant at *p* < 0.05000.

Variables	The Sum of a Rank for Diabetics Group	The Sum of a Rank for Cataract Group	U	Z	*p*	Z (Corrected for Ties)	P	2*1 P
KYNA	185.00	221.00	11.00	3.4836	0.0005	3.4836	0.0005	0.0001
KYN	161.00	245.00	35.00	2.2630	0.0236	2.2630	0.0236	0.0213
TRP	120.00	286.00	76.00	0.1780	0.8587	0.1780	0.8587	0.8617
TRP/KYNA	108.00	298.00	72.00	−0.3814	0.7029	−0.3814	0.7029	0.7086
TRP/KYN	71.00	335.00	35.00	−2.2630	0.0236	−2.2630	0.0236	0.0213
KYN/KYNA	150.00	256.00	46.00	1.7036	0.0885	1.7036	0.0885	0.0887
Age of patient	83.00	323.00	47.000	−1.6528	0.0984	−1.6562	0.0977	0.0991

U—the values of the Mann-Whitney test when the group size is less than 20, Z—Mann Whitney test value when the group size is greater than 20, *p*—probability value for the test for value of the test statistics Z, Z—test statistic Z (corrected for ties), P—probability value for test statistic Z (corrected for ties), 2*1 P—probability value when the group size is less than 20.

**Table 6:** Precursor ions and fragment ions of tryptophan, kynurenine, kynurenic acid and internal standards—mass spectrometry parameters.

Analyte	Precursor Ion (m/z)	Ion Transition	Declustering Potential	Collision Energy (eV)	Collision Cell Exit Potential	Entrance Potential
Tryptophan	205.0	188.0 146.0	50	16 23	16 10	10 10
Kynurenine	209.1	191.9 146.0	66	11 25	14 10	10 10
Kynurenic acid	190.0	172.0 144.0	81	17 25	12 14	10 10
(IS)	206.0	188.0	50	16	16	10
